# Developing Synergistic Relationships between Age-Friendly Universities and Age-Friendly Communities

**DOI:** 10.1093/geroni/igab046.374

**Published:** 2021-12-17

**Authors:** Joann Montepare, Wendy Rogers

## Abstract

The Age-Friendly University (AFU) initiative was designed to support the Global Network for Age-Friendly Cities and Communities (WHO, 2018) and offers a range of opportunities for institutions of higher education to help communities adapt to their new age-diverse social structures as a result of shifting age demographics. In turn, age-friendly community partnerships are helping to fuel campus efforts to advance age-inclusivity through education, research, and community engagement. At present over 70 institutions have joined the AFU global network, as more campuses prepare to become age-friendly partners. In this collaborative symposium (Directors of Aging Centers and AFU Interest Groups), campus leaders will describe synergistic relationships between their age-friendly campus efforts and the age-friendly efforts of their neighboring communities. Montepare (Lasell University) will provide an overview of the AFU initiative and its set of 10 principles, and make the case that campuses and communities are necessary partners for creating and developing age-friendly efforts. Demonstrating this assertion, Pastor and Rogers (University of Illinois Urbana-Champaign) will describe linkages between their community and campus initiatives, including developing a Panel of Elders, television programming for older adults, and hosting joint events. Black and Andel (University of South Florida) will discuss the intersection between the AFU principles and the processes undertaken by age-friendly communities. Revell and Viveiros (University of Massachusetts Dartmouth) will show how campus collaborations with nearby communities are instrumental in sustaining age-friendly efforts, especially during a pandemic.

